# Characteristics of Very Young Patients Undergoing Total Hip Arthroplasty: A Contemporary Assessment

**DOI:** 10.1016/j.artd.2023.101268

**Published:** 2023-12-28

**Authors:** Jennifer C. Wang, Kevin C. Liu, Brandon S. Gettleman, Matthew Chen, Amit S. Piple, Jaewon Yang, Nathanael D. Heckmann, Alexander B. Christ

**Affiliations:** a Keck School of Medicine of University of Southern California, Los Angeles, CA, USA; b University of South Carolina School of Medicine, Columbia, SC, USA; c University of Washington Medical Center, Seattle, WA, USA; d University of California Los Angeles, Los Angeles, CA, USA

**Keywords:** Total hip arthroplasty, Young patients, Indications, Complications

## Abstract

**Background:**

This study aims to compare indications, patient characteristics, hospital factors, and complication rates between total hip arthroplasty (THA) patients aged 30 years or younger and those older than 30 years using a large national database.

**Methods:**

The Premier Healthcare Database was utilized to identify primary THA patients from 2015 to 2021 who were aged ≤30 or >30 years. Patient demographics, hospital factors, and primary indications were compared for each cohort. Rates of complications and readmissions were assessed for each cohort by primary indication. Differences were assessed through univariate analysis.

**Results:**

Overall, 539,173 primary THA patients were identified (age ≤30: 1849; >30: 537,234). Compared to the >30 cohort, the ≤30 cohort was more likely to be male (56.5% vs 44.9%, *P* < .001) and non-White (34.0% vs 14.2%, *P* < .001). The most common indications for THA in the ≤30 cohort were osteonecrosis (49.3%), osteoarthritis (17.8%), and congenital hip deformities (16.0%), and in the >30 cohort, they were osteoarthritis (77.0%), other arthritis (11.3%), and osteonecrosis (5.4%). Patients aged ≤30 years had lower rates of respiratory failure (0.16% vs 0.57%, *P* < .001), acute renal failure (0.32% vs 1.72%, *P* < .001), and urinary tract infection (0.38% vs 1.11%, *P* = .003) than those aged >30 years, but higher rates of wound dehiscence (0.59% vs 0.29%, *P* = .015) and transfusion (3.68% vs 2.21%, *P* < .001). There were no differences in 90-day readmission rates (*P* = .811) or 90-day in-hospital death (*P* = .173) between cohorts.

**Conclusions:**

Younger patients undergoing THA differed significantly in indication, patient characteristics, and hospital factors compared to the older population on univariate analysis. Despite differences in indications, the cohorts did not differ markedly with regard to complication rates in this study.

## Introduction

Historically, surgeons have exhibited caution when considering total hip arthroplasty (THA) in young patients, primarily due to the concern for increased revision rates [1,2]. However, advances in implant materials and surgical techniques have decreased revision rates over time, and surgeons have become less reticent to indicate young patients for THA in the appropriate setting [[3], [4], [5], [6]]. Indications for THA in young patients, often defined as aged <30 or <50 in the literature, include symptomatic, irreparable cartilage loss, extensive bone loss, erosion of the acetabulum, and femoral head collapse [1,5,[7], [8], [9], [10], [11], [12]]. Kahlenberg et al. examined THAs performed in patients aged <21 years in the United States at a single institution and noted an increase in surgeries performed in this population from 347 in 2000 to 551 in 2016 [13]. Few large-scale epidemiological data on this subset of arthroplasty patients are available, particularly with contemporary data.

Young patients with a complex hip disease are increasingly willing to undergo THA to maintain desired levels of function and reduce pain [3]. While there is extensive literature regarding rates of revision in the young population [2,5,14], there is a paucity of data examining rates of specific complications among young THA patients—questions important in guiding patient counseling, perioperative care, and clinical research [1]. Therefore, this study aims to assess for differences in the indications, patient characteristics, hospital factors, and complication rates of patients aged ≤30 years and >30 years who underwent primary THA.

## Material and methods

The Premier Healthcare Database [15] was queried for all patients who underwent primary, elective THA from January 1, 2015, to December 31, 2021. The Premier Healthcare Database is an all-payer claims database containing patient and hospital characteristics, medications, International Classification of Diseases (ICD) diagnosis and procedural codes, Current Procedural Terminology codes, and patient-specific billing information from approximately 25% of all United States hospital admissions. ICD-10 procedural and Current Procedural Terminology codes were utilized to identify these procedures [15]. Those who underwent THA for nonprimary, nonelective indications were excluded (Supplemental Table 1). This study was exempt from institutional review board approval as all information was de-identified in accordance with the Health Insurance Portability and Accountability Act.

### Identification of study cohorts

Two subsets of primary, elective THA patients were identified: age ≤30 years (“≤30 cohort”) and age >30 years (“>30 cohort”) at the time of index surgery.

**Figure 1:**
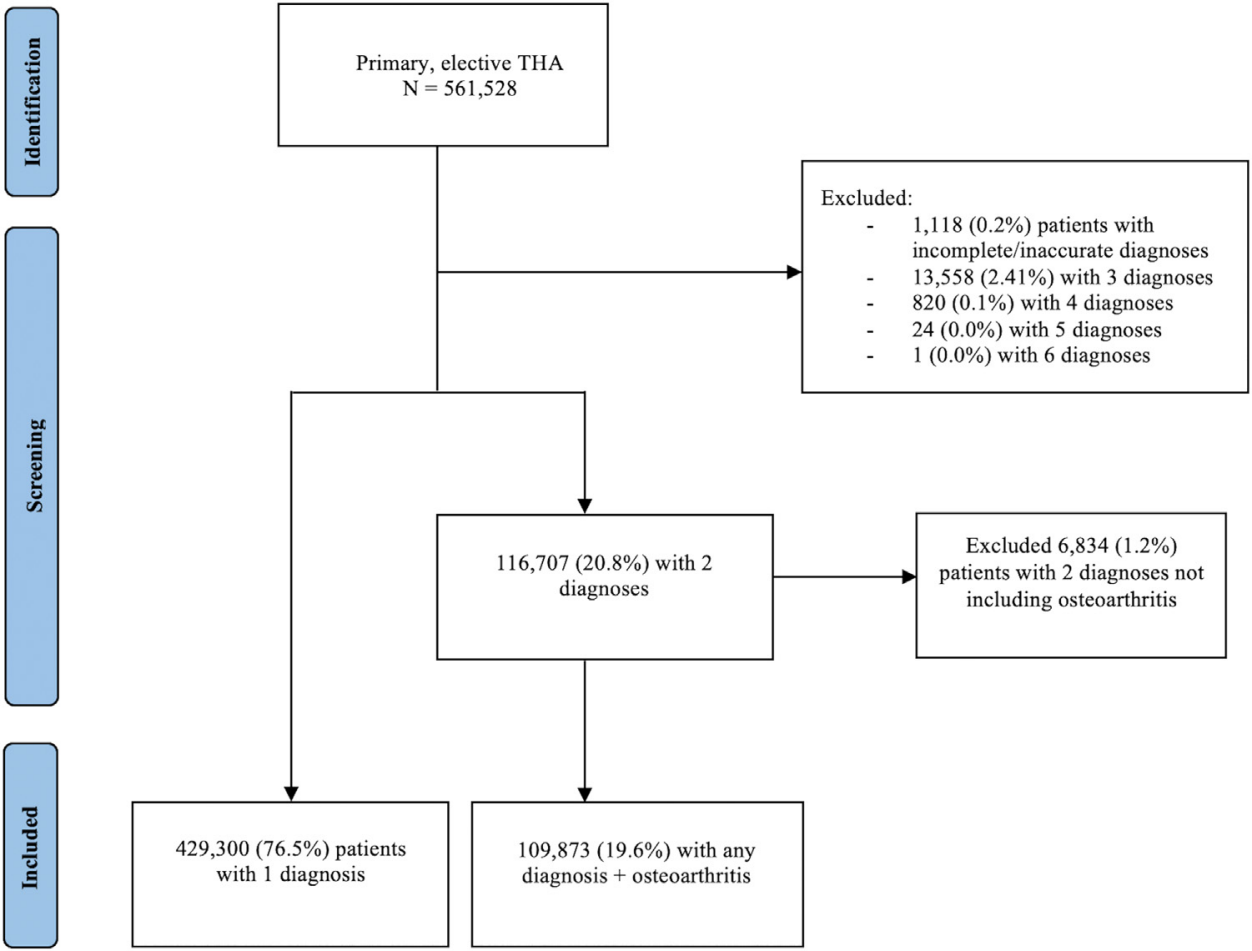
Flowchart describing final cohort selection. Abbreviation: THA, total hip arthroplasty.

Using ICD-10 diagnosis codes, patients were categorized by the primary indication for THA (ie, osteoarthritis [OA], osteonecrosis, posttraumatic arthritis, neoplasm, congenital hip deformity, inflammatory/infectious, and other arthritis; Supplemental Table 2). For patients meeting 2 indications, the indication other than OA was considered as the primary diagnosis as it would likely represent the most immediate cause for surgery. For example, if an ICD-10 diagnosis code for inflammatory arthritis and OA were present, we assumed the patient underwent THA due to the inflammatory process. This methodology has been performed by Yakkanti et al. [16]. Patients meeting 2 indications other than OA (1.2%) or meeting >2 indications (2.5%) were excluded from this analysis as these scenarios precluded the ability to identify the most likely indication for surgery reliably. Patients with miscoded (eg, arthritis of the knee) or nonspecific (eg, orthopedic aftercare, presence of orthopedic joint implants, and so on) diagnosis codes (0.2%) were also excluded from the analysis. In total, 561,528 primary, elective THA patients were identified with 539,173 (96.0%) remaining after applying the exclusion criteria (Fig. 1).

### Independent variables

Patient demographics (ie, age, sex, race, length of stay, year of procedure, and total cost), hospital factors (ie, size, urban/rural setting, teaching status, and region), and the prevalence of patient comorbidities were assessed and compared between the ≤30 and >30 cohorts.

### Study endpoints

Outcomes assessed were the 90-day rates of prosthetic complications (dislocation, mechanical loosening, periprosthetic fracture, periprosthetic joint infection), thromboembolic complications (deep vein thrombosis and pulmonary embolism, stroke, myocardial infarction), bleeding-related events (acute blood loss anemia, hemarthrosis, hemorrhage, transfusion), infectious complications (surgical site infection, sepsis), wound complications (wound dehiscence, seroma, hematoma), medical complications (pneumonia, respiratory failure, acute renal failure, urinary tract infection), readmission, and in-hospital death.

### Statistical analysis

All patient demographics, hospital factors, comorbidities, and postoperative complications were evaluated with descriptive statistics. In order to assess differences between the ≤30 and >30 cohorts, independent *t*-tests were performed for continuous variables while Fischer’s exact or chi-squared tests were utilized for categorical variables where appropriate. Statistical significance was defined as *P* < .050. All statistical analyses were performed using STATA (version 16.1; StataCorp, College Station, TX).

## Results

### Differences in patient and hospital characteristics

Of the 539,173 patients who underwent a primary THA from 2015 to 2021, 1849 (0.3%) were ≤30 years old (mean: 25.7 ± 4.0 years), and 537,324 (99.7%) were >30 years old (mean: 65.9 ± 10.4 years). More male (56.5% vs 44.9%, *P* < .001) and fewer White patients (66.0% vs 85.8%, *P* < .001) comprised the ≤30 cohort compared to the >30 cohort. The ≤30 cohort had a greater proportion of patients who were treated at a large hospital (>500 beds: 38.3% vs 24.8%, *P* < .001) and at a teaching institution (57.9% vs 45.3%, *P* < .001) than the >30 cohort. Compared to the >30 cohort, the ≤30 cohort had a significantly longer length of stay (1.7 ± 2.0 vs 1.6 ± 1.4 days, *P* = .034). Finally, the ≤30 cohort also had a higher cost of treatment than the >30 cohort ($17,267.14 ± 8582.98 vs $15,612.08 ± 9379.31, *P* = .001; Table 1).

**Table 1:** Patient demographics and hospital characteristics.

Variable	≤30 Cohort, N = 1849	>30 Cohort, N = 537,324	*P* value
	Mean ± standard deviation	Mean ± standard deviation	
Age (y)	25.7 ± 4.0	65.9 ± 10.4	<.001
Length of stay (d)	1.7 ± 2.0	1.6 ± 1.4	.0338
Total cost ($)	17,267.14 ± 8582.98	15,612.08 ± 9379.31	<.001

Variable	N	%	N	%	
Gender					
Male	1045	56.5	241,469	44.9	<.001
Female	804	43.5	295,821	55.1	
Race					
Asian	40	2.2	4251	0.8	<.001
Black	359	19.4	43,688	8.1	
Other	161	8.7	21,035	3.9	
Unknown	69	3.7	7373	1.4	
Caucasian	1220	66.0	460,977	85.8	
Marital status					
Married	339	18.3	305,283	56.8	<.001
Other	171	9.2	42,314	7.9	
Single	1331	72.0	187,746	34.9	
Bed size					
<100	147	8.0	47,422	8.8	<.001
100-199	290	15.7	103,330	19.2	
200-299	276	14.9	104,069	19.4	
399-399	273	14.8	87,820	16.3	
400-499	155	8.4	61,387	11.4	
>500	708	38.3	133,296	24.8	
Urban vs Rural					
Rural	148	8.0	57,867	10.8	<.001
Urban	1701	92.0	479,457	89.2	
Teaching status					
No	779	42.1	294,128	54.7	<.001
Yes	1070	57.9	243,196	45.3	
Region					
Midwest	381	20.6	137,497	25.6	<.001
Northeast	321	17.4	98,416	18.3	
South	878	47.5	218,716	40.7	
West	269	14.5	82,695	15.4	

### Differences in primary diagnoses

The top 3 indications for THA in the ≤30 cohort were osteonecrosis (49.3%), OA (17.8%), and congenital hip deformities (16.0%). The top 3 indications in the >30 cohort were OA (77.0%), other arthritis (11.3%), and osteonecrosis (5.4%; Table 2).

**Table 2:** Indications for total hip arthroplasty by age group.

Indication	≤30 Cohort, N = 1849		>30 Cohort, N = 537,324		Total
	N	%	N	%	
Osteoarthritis	330	17.8	413,926	77.0	414,256
Osteonecrosis	911	49.3	29,117	5.4	60,028
Post-traumatic arthritis	99	5.4	6846	1.3	6945
Neoplasm	12	0.6	625	0.1	637
Congenital	296	16.0	7215	1.3	7511
Infectious	6	0.3	121	0.0	127
Inflammatory	105	5.7	18,849	3.5	18,954
Other arthritis	90	4.9	60,625	11.3	60,715
Total	1849	100.0	537,324	100.0	539,173

### Differences in comorbidities

Of the 33 comorbidities investigated, there were 23 with rates that significantly differed between the cohorts. The ≤30 cohort had significantly lower rates of hemiplegia/paraplegia (0.54% vs 0.08%, *P* < .001), other neurological disorders (3.03% vs 1.95%, *P* = .001), rheumatic disease (6.81 vs 3.13, *P* < .001), HIV/AIDS (0.49% vs 0.11%, *P* < .001), weight loss (0.59% vs 0.25%, *P* = .003), alcohol abuse (2.11% vs 1.26%, *P* = .001), drug abuse (4.38% vs 1.19%, *P* < .001), and psychoses (0.49% vs 0.22%, *P* = .012; Table 3).

**Table 3:** Patient comorbidities.

Comorbidity	≤30 Cohort, N = 1849		>30 Cohort, N = 537,324		*P* value
	N	%	N	%	
Congestive heart failure	11	0.59	19,449	3.62	<.001
Myocardial infarction	5	0.27	19,838	3.69	<.001
Valvular disease	5	0.27	3930	0.73	.020
Pulmonary hypertension	9	0.49	3485	0.65	.387
Chronic pulmonary disease	240	12.98	78,767	14.66	.042
Peripheral vascular disease	4	0.22	14,605	2.72	<.001
Hypertension	186	10.06	287,885	53.58	<.001
Complicated hypertension	25	1.35	41,681	7.76	<.001
Cerebrovascular accident	31	1.68	21,429	3.99	<.001
Hemiplegia/Paraplegia	10	0.54	453	0.08	<.001
Other neurological disorders	56	3.03	10,478	1.95	.001
Diabetes, uncomplicated	34	1.84	61,073	11.37	<.001
Diabetes, complicated	6	0.32	25,139	4.68	<.001
Hypothyroidism	70	3.79	80,060	14.90	<.001
Renal failure	35	1.89	34,747	6.47	<.001
Liver disease	20	1.08	6974	1.30	.412
Chronic peptic ulcer disease	1	0.05	1313	0.24	.098
Blood loss anemia	6	0.32	3115	0.58	.149
Deficiency anemia	18	0.97	6504	1.21	.352
Coagulopathy	43	2.33	10,353	1.93	.213
Venous thromboembolism	60	3.24	23,501	4.37	.018
Fluid and electrolyte disorders	56	3.03	30,025	5.59	<.001
Rheumatic disease	126	6.81	16,841	3.13	<.001
HIV/AIDS	9	0.49	608	0.11	<.001
Lymphoma	5	0.27	1189	0.22	.654
Solid tumor	11	0.59	2950	0.55	.790
Metastatic cancer	2	0.11	730	0.14	.747
Obesity	294	15.90	128,589	23.93	<.001
Weight loss	11	0.59	1347	0.25	.003
Alcohol abuse	39	2.11	6777	1.26	.001
Drug abuse	81	4.38	6371	1.19	<.001
Psychoses	9	0.49	1159	0.22	.012
Depression	212	11.47	66,825	12.44	.207

### Differences in postoperative outcomes

The ≤30 cohort had statistically significant greater rates of wound dehiscence (0.59% vs 0.29%, *P* = .015) and transfusion (3.68% vs 2.21%, *P* < .001) but lower rates of respiratory failure (0.16% vs 0.57%, *P* = .019), acute renal failure (0.32% vs 1.72%, *P* < .001), and urinary tract infection (0.38% vs 1.11%, *P* = .003) than the >30 cohort (Table 4). There were no differences in 90-day readmission rates (3.08% vs 2.99%, *P* = .811) and 90-day in-hospital death (0.00% vs 0.10%, *P* = .173) between the cohorts. There was also no difference in the rates of dislocation (0.49% vs 0.61%, *P* = .511), periprosthetic fracture (0.05% vs 0.29%, *P* = .062), implant loosening (0.00% vs 0.05%, *P* = .329), periprosthetic joint infection (0.76% vs 0.57%, *P* = .273), surgical site infection (0.16% vs 0.12%, *P* = .603), and acute blood loss anemia (15.7% vs 13.4%, *P* = .065) although the ≤30 group did have a significantly increased rate of blood transfusion (3.68% vs 2.21%, *P* < .001). Differences in postoperative outcomes by indication for surgery are available in Supplemental Table 3.

**Table 4:** Overall complication rate by cohort.

Complication	≤30 Cohort, N = 1849		>30 Cohort, N = 537,324		*P* value
	N	%	N	%	
Hip dislocation	9	0.49	3254	0.61	.511
Hip prosthetic loosening	0	0.00	277	0.05	.329
Periprosthetic fracture	1	0.05	1535	0.29	.062
Periprosthetic joint infection	14	0.76	3039	0.57	.273
Sepsis	6	0.32	1589	0.30	.82
Surgical site infection	3	0.16	646	0.12	.603
Pulmonary embolism	3	0.16	1125	0.21	.658
Deep vein thrombosis	5	0.27	1744	0.32	.683
Wound dehiscence	11	0.59	1558	0.29	**.015**
Seroma	1	0.05	402	0.07	.745
Stroke	0	0.00	600	0.11	.151
Pneumonia	8	0.43	1450	0.27	.178
Respiratory failure	3	0.16	3084	0.57	**.019**
Myocardial infarction	0	0.00	858	0.16	.085
Acute renal failure	6	0.32	9241	1.72	**<.001**
Urinary tract infection	7	0.38	5991	1.11	**.003**
Hematoma	7	0.38	1334	0.25	.261
Hemarthrosis	0	0.00	39	0.01	.714
Acute blood loss anemia	291	15.74	93,316	17.37	.065
Hemorrhage	3	0.16	657	0.12	.624
Transfusion	68	3.68	11,861	2.21	**<.001**
90-d readmission	57	3.08	16,055	2.99	.811
90-d in-hospital death	0	0.00	540	0.10	.173

Bold values indicate statistical significance.

## Discussion

While THA is more commonly performed in older adults, an increasing number of young people undergo this procedure. This patient population will continue to grow as both patient and surgeon confidence in the durability of THA increases over time. However, contemporary data characterizing the postoperative outcomes of this unique population are lacking. In this study, patients aged ≤30 years were more likely to be non-White and male, had osteonecrosis as the most common indication for surgery, and had minimal differences in rates of 90-day postoperative complications when compared to patients aged >30 years.

Existing data on the indications for THA among young arthroplasty patients are limited by study heterogeneity. Prior studies have used various age cutoffs including <50 years [[17], [18], [19], [20], [21], [22]], ≤35 years [[23], [24], [25], [26], [27], [28], [29], [30], [31]], and ≤25 years [[32], [33], [34]]. Thirty years of age was utilized as the age cutoff presently as that is what has been used most frequently to define a very young arthroplasty population [[23], [24], [25], [26],^28^]. However, examining age beyond a binary variable may be of interest to future investigations. Of studies that utilized a similar age cutoff as the present study, none included patients who underwent surgery beyond the first decade of the 21st century [[23], [24], [25], [26],^28^]. As operative techniques, perioperative care, and implant design continue to improve, contemporary data on outcomes in this unique patient population are necessary to further guide these efforts and potentially expand indications to help more patients in need. The data presented here represent the most contemporary data published on indications and outcomes in patients aged <30 years who underwent a primary, elective THA in the United States.

While inflammatory arthropathies comprised the most commonly studied and most common indication for THA in young patients in the 1900s and early 2000s [23,29,30,35], more contemporary studies report higher numbers of patients aged ≤35 years who underwent THA for congenital dysplasia [24], osteonecrosis [25], or OA secondary to pediatric hip disease [26]. In our study, most patients aged <30 years underwent THA from 2015 to 2021 for osteonecrosis (49.3%), which corroborates the data presented by Girard et al. [25]. These data demonstrate that the primary etiology for which young patients are seeking out THA has changed, likely owing to advancements in the medical management of rheumatologic diseases, which may be relevant in longitudinal studies on implant survivorship in this population.

In the present study, low rates of 90-day medical and surgical complications in patients aged ≤30 years were observed regardless of the indication for THA. Most existing studies defined revision and radiographic loosening as the endpoints of interest [23,29,31] and less have commented on other medical and surgical complications [[24], [25], [26],^28^,^30^]. None have examined outcomes stratified by index diagnosis as reported in this study. We observed minimal differences in postoperative complication rates between patients aged ≤30 years and >30 years by surgical indication. Understanding the types of patients that undergo THA and the outcomes experienced by this population is important to patient counseling, resource allocation, and perioperative care optimization.

The data presented identify sociodemographic differences that warrant discussion. In this study, patients aged ≤30 years who underwent primary, elective THA were more likely to be non-White, male, and from the South than their >30-year-old counterparts. These observations may be explained in part by epidemiologic differences of various hip diseases, such as avascular necrosis secondary to sickle cell [36], hip fracture [37], and slipped capital femoral epiphysis [38]. The greater proportion of White patients in the >30-year-old cohort may also be explained by a higher total joint arthroplasty utilization rate among Medicare-eligible White patients than that among non-White patients [39]. Prior studies examining the young arthroplasty population did not comment on the racial breakdown of their respective study cohorts.

A number of limitations to this study require acknowledgment. First, as a retrospective study, it is reliant on the accuracy of administrative coding. However, we believe that in the absence of systematically biased errors, the accuracy of the data reported from a comparative standpoint is unlikely to be compromised. Second, this study examines a ≤30 cohort although different definitions of “young” regarding THA patients have been used in the literature. We elected to use this cutoff as (1) this is what has been used most commonly by the orthopedic community [[23], [24], [25], [26],^28^] and (2) this cohort may have differences in baseline health and functional demands compared to even those aged <40 and <50 years, in addition to Medicare-eligible patients. Third, it must be noted that a multivariate analysis was not performed. However, as the purpose of the study was to describe contemporary data on young and old THA patients, we do not believe this is a critical limitation but rather aligns with the study design. Further elucidating whether differences between the 2 age groups exist after accounting for confounders and the clinical implications of that is a pursuit that would warrant a separate study.

There are numerous strengths to this study that warrant mention. First, to the best of our knowledge, this study comprises the largest sample of young patients undergoing primary, elective THA in the United States. Young patients are challenging to study due to the relatively low number of THAs performed in this population, and the relatively rare incidence of complications associated with elective THAs precludes robust studies in this unique but important population. This crucial limitation in the prior literature is addressed by the present study. Second, existing studies on this subset of patients utilize heterogeneous study cohorts in which many disease etiologies are aggregated for analysis. Furthermore, few studies discuss the outcomes of THA in young patients with noninflammatory hip degeneration. In the present study, we examined common hip disease etiologies independently, providing data that can be more accurately applied in clinical settings. The data in this study also demonstrate the importance of future clinical studies to focus on patients with noninflammatory diseases, as this is now the most common etiology for THA in the young arthroplasty population.

## Conclusions

In this study, we identified 1849 patients aged ≤30 years and 537,234 patients aged >30 years who underwent primary, elective THA from 2015 to 2020. Patients aged ≤30 years were more likely to be non-White and male and carry a primary diagnosis of osteonecrosis as the indication for THA. Young patients had minimal differences in 90-day postoperative complications when compared to patients aged >30 years. The study comprises the largest sample of young patients undergoing primary, elective THA in the United States, an increasingly relevant patient population as implant longevity and surgical care continue to improve.

## Conflicts of interest

D. Heckmann receives royalties from Corin U.S.A. D. Heckmann is a paid consultant for ntellijoint Surgical, MicroPort Orthopedics, Corin U.S.A, Zimmer. D. Heckmann owns stock or stock options in Intellijoint Surgical. D. Heckmann is a board member for AAOS, AJRR, AAHKS; B. Christ is a paid consultant for Intellijoint Surgical, Smith & Nephew. B. Christ is a board member for AAOS, Musculoskeletal Tumor Society, Orthopaedic Research Society; all other authors declare no potential conflicts of interest.

For full disclosure statements refer to https://doi.org/10.1016/j.artd.2023.101268.

## Author contributions

K.C.L., B.S.G., and J.C.W. contributed to data curation. All authors contributed to formal analysis, designing the study methodology, and reviewing and editing the article. K.C.L., B.S.G., J.C.W., A.S.P., J.Y., and M.C. carried out investigation and contributed to writing the original draft. J.C.W. contributed to conceptualization. N.D.H. and A.B.C. contributed to supervision.

## Figures and Tables

**Supplemental Table 1:** Excluded ICD-10 and CPT codes.

ICD-10 codes	Description
Total joint arthroplasty	Total knee arthroplasty: 0SRC0- + 0SRD0-
Trauma	M80.05, M80.06, M80.85, M80.86, M84.35, M84.36, M84.45, M84.46, M84.65, M84.66, M84.75, S32.3, S32.4, S32.5, S32.6, S32.8, S72, S79.0, S79.1, S82.0, S82.1, S82.2, S82.3, S82.4, S82.8, S82.9, S89.0, S89.1, S89.2, S89.3, M96.65, M96.66, M96.67, M96.69
Periprosthetic fracture	M97.0, M97.1
Complication of orthopaedic implants	T84.0, T84.116, T85.117, T84.124, T84.125, T84.126, T84.127, T84.194, T84.195, T84.196, T84.197, T84.218, T84.228, T84.3, T84.4, T84.8, T84.9

CPT codes	Description
Total joint arthroplasty	Total knee arthroplasty: 27447

CPT, Current Procedural Terminology.

**Supplemental Table 2:** ICD-10 diagnosis codes for indications of primary, elective THA.

Group 1: Primary OA	M15.0- , M16.0-, M16.1-
Group 2: Osteonecrosis	D57-, M87.0-, M87.1-, M87.3-, M87.8-, M87.9-, M90.55-, M94.3-
Group 3: Posttraumatic	M87.2-, M16.4-, M16.5-, M12.5-, S79.0-, M21-, M95.5-
Group 4: Neoplasms	M12.25-, C40-, C41-, C49-, C76.5-, C79.5-, D16.2-, D16.9-, D48.0-, D49-
Group 5: Congenital	M16.2-, M16.3-, M91.1-, M93.0-, Q65-, Q74.2-, Q77-
Group 6: Inflammatory/infectious	L40, M00-, M01-, M02-, M04-, M05-, M06-, M07-, M12.0-, M32-, M35-, M08.0-, M08.1-, M08.2-, M08.3-, M08.4-
Group 7: Other arthritis	M13-, M19-, M24-, M25-, M89.9-, M16.6-, M16.7-, M16.9-, M08.8-, M08.9-, M12.8-, M89.8X-, M93.8-, M15.1-, M15.2-, M15.3-, M15.4-, M15.8-, M15.9-

**Supplemental Table 3:** Complications by surgical indication.

Complication	Osteoarthritis only				*P*-value
	≤ 30 y old N = 330		> 30 y old N = 413,926		
	N	%	N	%	
Hip dislocation	2	0.61	2271	0.55	.888
Hip prosthetic loosening	0	0.00	187	0.05	.699
Periprosthetic fracture	0	0.00	1140	0.28	.340
Periprosthetic joint infection	2	0.61	2152	0.52	.828
Sepsis	2	0.61	1162	0.28	.264
Surgical site infection	0	0.00	438	0.11	.554
Pulmonary embolism	0	0.00	836	0.20	.414
Deep vein thrombosis	1	0.30	1276	0.31	.986
Wound dehiscence	3	0.91	1102	0.27	.024
Seroma	0	0.00	299	0.07	.625
Stroke	0	0.00	468	0.11	.541
Pneumonia	1	0.30	1012	0.24	.830
Respiratory failure	0	0.00	2206	0.53	.184
Myocardial infarction	0	0.00	642	0.16	.474
Acute renal failure	1	0.30	6876	1.66	.054
Urinary tract infection	1	0.30	4463	1.08	.173

Complication	Osteonecrosis only				*P*-value
	≤ 30 y old N = 911		> 30 y old N = 29,117		
	N	%	N	%	
Hip dislocation	3	0.33	283	0.97	.049
Hip prosthetic loosening	0	0.00	27	0.09	.358
Periprosthetic fracture	1	0.11	80	0.27	.344
Periprosthetic joint infection	10	1.10	224	0.77	.267
Sepsis	2	0.22	116	0.40	.396
Surgical site infection	3	0.33	55	0.19	.342
Pulmonary embolism	2	0.22	71	0.24	.883
Deep vein thrombosis	3	0.33	104	0.36	.889
Wound dehiscence	8	0.88	109	0.37	.016
Seroma	0	0.00	26	0.09	.367
Stroke	0	0.00	43	0.15	.246
Pneumonia	5	0.55	149	0.51	.877
Respiratory failure	2	0.22	317	1.09	.012
Myocardial infarction	0	0.00	46	0.16	.230
Acute renal failure	5	0.55	652	2.24	.001
Urinary tract infection	2	0.22	356	1.22	.006
Hematoma	4	0.44	85	0.29	.421
Hemarthrosis	0	0.00	1	0.00	.860
Acute blood loss anemia	129	14.16	5416	18.60	.001
Hemorrhage	2	0.22	58	0.20	.892
Transfusion	46	5.05	1002	3.44	.009
90-d readmission	36	3.95	1134	3.89	.930
90-d in-hospital death	0	0.00	380	1.31	.275

Complication	Posttraumatic arthritis				*P*-value
	≤ 30 y old N = 99		> 30 y old N = 6846		
	N	%	N	%	
Hip dislocation	2	2.02	77	1.12	.404
Hip prosthetic loosening	0	0.00	6	0.09	.768
Periprosthetic fracture	0	0.00	23	0.34	.563
Periprosthetic joint infection	0	0.00	61	0.89	.345
Sepsis	0	0.00	24	0.35	.555
Surgical site infection	0	0.00	9	0.13	.718
Pulmonary embolism	1	1.01	16	0.23	.121
Deep vein thrombosis	0	0.00	39	0.57	.451
Wound dehiscence	0	0.00	23	0.34	.563
Seroma	0	0.00	7	0.10	.750
Stroke	0	0.00	7	0.10	.750
Pneumonia	0	0.00	25	0.37	.547
Respiratory failure	0	0.00	57	0.83	.362
Myocardial infarction	0	0.00	14	0.20	.652
Acute renal failure	0	0.00	145	2.12	.143
Urinary tract infection	0	0.00	90	1.31	.251
Hematoma	0	0.00	28	0.41	.524
Hemarthrosis	0	0.00	1	0.01	.904
Acute blood loss anemia	21	21.21	1423	20.79	.917
Hemorrhage	0	0.00	9	0.13	.718
Transfusion	2	2.02	252	3.68	.382
90-d readmission	0	0.00	272	3.97	.930
90-d in-hospital death	0	0.00	8	0.12	.275

Complication	Neoplasm				*P*-value
	≤ 30 y old N = 12		> 30 y old N = 625		
	N	%	N	%	
Hip dislocation	0	0.00	12	1.92	.628
Hip prosthetic loosening	0	0.00	1	0.16	.890
Periprosthetic fracture	0	0.00	5	0.80	.756
Periprosthetic joint infection	0	0.00	5	0.80	.756
Sepsis	0	0.00	10	1.60	.659
Surgical site infection	0	0.00	1	0.16	.890
Pulmonary embolism	0	0.00	9	1.44	.675
Deep vein thrombosis	0	0.00	10	1.60	.659
Wound dehiscence	0	0.00	5	0.80	.756
Seroma	0	0.00	2	0.32	.844
Stroke	0	0.00	4	0.64	.781
Pneumonia	1	8.33	8	1.28	.040
Respiratory failure	1	8.33	15	2.40	.193
Myocardial infarction	0	0.00	2	0.32	.844
Acute renal failure	0	0.00	41	6.56	.359
Urinary tract infection	0	0.00	12	1.92	.628
Hematoma	0	0.00	3	0.48	.810
Hemarthrosis	0	0.00	0	0.00	--
Acute blood loss anemia	2	16.67	198	31.68	.267
Hemorrhage	0	0.00	0	0.00	--
Transfusion	1	8.33	57	9.12	.925
90-d readmission	2	16.67	47	7.52	.239
90-d in-hospital death	0	0.00	7	1.12	.712

Complication	Congenital				*P*-value
	≤ 30 y old N = 296		> 30 y old N = 7215		
	N	%	N	%	
Hip dislocation	1	0.34	46	0.64	.522
Hip prosthetic loosening	0	0.00	1	0.01	.839
Periprosthetic fracture	0	0.00	18	0.25	.390
Periprosthetic joint infection	0	0.00	36	0.50	.223
Sepsis	1	0.34	13	0.18	.538
Surgical site infection	0	0.00	7	0.10	.592
Pulmonary embolism	0	0.00	16	0.22	.417
Deep vein thrombosis	0	0.00	20	0.28	.364
Wound dehiscence	0	0.00	14	0.19	.448
Seroma	0	0.00	2	0.03	.775
Stroke	0	0.00	3	0.04	.726
Pneumonia	0	0.00	14	0.19	.448
Respiratory failure	0	0.00	17	0.24	.403
Myocardial infarction	0	0.00	8	0.11	.567
Acute renal failure	0	0.00	80	1.11	.069
Urinary tract infection	1	0.34	57	0.79	.384
Hematoma	0	0.00	11	0.15	.501
Hemarthrosis	0	0.00	0	0.00	--
Acute blood loss anemia	51	17.23	1517	21.03	.115
Hemorrhage	1	0.34	10	0.14	.380
Transfusion	6	2.03	242	3.35	.210
90-d readmission	7	2.36	159	2.20	.853
90-d in-hospital death	0	0.00	5	0.07	.651

Complication	Infectious				*P*-value
	≤ 30 y old N =6		> 30 y old N = 121		
	N	%	N	%	
Hip dislocation	0	0.00%	2	1.65%	1
Hip prosthetic loosening	0	0.00%	0	0.00%	-
Periprosthetic fracture	0	0.00%	0	0.00%	-
Periprosthetic joint infection	0	0.00%	6	4.96%	1
Sepsis	0	0.00%	2	1.65%	1
Surgical site infection	0	0.00%	2	1.65%	1
Pulmonary embolism	0	0.00%	0	0.00%	-
Deep vein thrombosis	0	0.00%	0	0.00%	-
Wound dehiscence	0	0.00%	1	0.83%	-
Seroma	0	0.00%	0	0.00%	-
Stroke	0	0.00%	0	0.00%	-
Pneumonia	0	0.00%	0	0.00%	-
Respiratory failure	0	0.00%	2	1.65%	1
Myocardial infarction	0	0.00%	0	0.00%	-
Acute renal failure	0	0.00%	8	6.61%	1
Urinary tract infection	0	0.00%	0	0.00%	-
Hematoma	0	0.00%	1	0.83%	1
Hemarthrosis	0	0.00%	0	0.00%	-
Acute blood loss anemia	1	16.67%	32	26.45%	1
Hemorrhage	0	0.00%	0	0.00%	-
Transfusion	0	0.00%	14	11.57%	1
90-d readmission	0	0.00%	12	9.92%	1
90-d in-hospital death	0	0.00%	0	0.00%	-

Complication	Inflammatory				*P*-value
	≤ 30 y old N =105		> 30 y old N = 18,849		
	N	%	N	%	
Hip dislocation	1	0.95%	194	1.03%	1
Hip prosthetic loosening	0	0.00%	17	0.09%	1
Periprosthetic fracture	0	0.00%	68	0.36%	1
Periprosthetic joint infection	1	0.95%	178	0.94%	1
Sepsis	0	0.00%	73	0.39%	1
Surgical site infection	0	0.00%	45	0.24%	1
Pulmonary embolism	0	0.00%	50	0.27%	1
Deep vein thrombosis	0	0.00%	88	0.47%	1
Wound dehiscence	0	0.00%	95	0.50%	1
Seroma	1	0.95%	18	0.10%	.1
Stroke	0	0.00%	14	0.07%	1
Pneumonia	0	0.00%	67	0.36%	1
Respiratory failure	0	0.00%	155	0.82%	1
Myocardial infarction	0	0.00%	40	0.21%	1
Acute renal failure	0	0.00%	394	2.09%	.285
Urinary tract infection	1	0.95%	285	1.51%	1
Hematoma	1	0.95%	60	0.32%	.288
Hemarthrosis	0	0.00%	0	0.00%	-
Acute blood loss anemia	22	20.95%	3895	20.66%	.904
Hemorrhage	0	0.00%	30	0.16%	1
Transfusion	6	5.71%	617	3.27%	.162
90-d readmission	3	2.86%	762	4.04%	.802

Complication	Other arthritis				*P*-value
	≤ 30 y old N = 90		> 30 y old N = 60,625		
	N	%	N	%	
Hip dislocation	0	0.00	369	0.61	.458
Hip prosthetic loosening	0	0.00	38	0.06	.812
Periprosthetic fracture	0	0.00	201	0.33	.584
Periprosthetic joint infection	1	1.11	377	0.62	.555
Sepsis	1	1.11	189	0.31	.175
Surgical site infection	0	0.00	89	0.15	.716
Pulmonary embolism	0	0.00	127	0.21	.664
Deep vein thrombosis	1	1.11	207	0.34	.212
Wound dehiscence	0	0.00	209	0.34	.577
Seroma	0	0.00	48	0.08	.789
Stroke	0	0.00	61	0.10	.763
Pneumonia	1	1.11	175	0.29	.147
Respiratory failure	0	0.00	315	0.52	.493
Myocardial infarction	0	0.00	106	0.17	.691
Acute renal failure	0	0.00	1045	1.72	.209
Urinary tract infection	2	2.22	728	1.20	.374
Hematoma	2	2.22	154	0.25	<.001
Hemarthrosis	0	0.00	22	0.04	.857
Acute blood loss anemia	19	21.11	11,280	18.61	.542
Hemorrhage	0	0.00	82	0.14	.727
Transfusion	3	3.33	1420	2.34	.535
90-d readmission	1	1.11	2035	3.36	.237
90-d in-hospital death	0	0.00	74	0.12	.740

## Appendix A Supplementary data

Conflict of Interest Statement for Christ

Conflict of Interest Statement for Piple

Conflict of Interest Statement for Wang

Conflict of Interest Statement for Heckmann

Conflict of Interest Statement for Gettleman

Conflict of Interest Statement for Yang

Conflict of Interest Statement for Liu

Conflict of Interest Statement for Chen
