# Supplementary material for: Characteristics of Very Young Patients Undergoing Total Hip Arthroplasty: A Contemporary Assessment
Source: Arthroplast Today. 2023 Dec 28;25:101268. doi: 10.1016/j.artd.2023.101268 (PMC10792167; doi:10.1016/j.artd.2023.101268)
Supplement: Conflict of Interest Statement for Gettleman [file mmc5.pdf]

DocuSign Envelope ID: E6B55F55-9248-40BE-A40F-320550184DDF

INDIVIDUAL CONFLICT OF INTEREST STATEMENT

American Association of Hip and Knee Surgeons

(Adopted from the American Academy of Orthopaedic Surgeons disclosure statement)

The following form **must be filled out completely and submitted by each author (example, 6 authors, 6 forms). All items require a response. If there is no relevant disclosure for a given item, enter "None."**

Manuscript Title: : Characteristics of Very Young Patients Undergoing Total Hip Arthroplasty

1. Royalties from a company or supplier (The following conflicts were disclosed)
None.

2. Speakers bureau/paid presentations for a company or supplier (The following conflicts were disclosed)
None.

3A. Paid employee for a company or supplier (The following conflicts were disclosed)
None.

3B. Paid consultant for a company or supplier (The following conflicts were disclosed)
None.

3C. Unpaid consultants for a company or supplier (The following conflicts were disclosed)
None.

4. Stock or stock options in a company or supplier (The following conflicts were disclosed)
None.

5. Research support from a company or supplier as a Principal Investigator (The following conflicts were disclosed)
None.

6. Other financial or material support from a company or supplier (The following conflicts were disclosed)
None.

7. Royalties, financial or material support from publishers (The following conflicts were disclosed)
None.

8. Medical/Orthopaedic publications editorial/governing board (The following conflicts were disclosed)
None.

9. Board member/committee appointments for a society (The following conflicts were disclosed)
None.

<u>Each author must sign AND print or type his/her name, date and submit a separate form</u>

In addition, one BLINDED Conflict of Interest form (no author names used) should be submitted per manuscript with all author disclosures.

Brandon Gettleman	DocuSigned by: Brandon Gettleman 9876CE8E6C644C7...	3/28/23
Author Name (Print or Type)	Author Signature	Date
